# How to Work Collaboratively Within the Health System: Workshop Summary and Facilitator Reflection

**DOI:** 10.15171/ijhpm.2019.131

**Published:** 2019-12-08

**Authors:** Christine E. Cassidy, Sarah Bowen, Guillaume Fontaine, Élizabeth Côté-Boileau, Ingrid Botting

**Affiliations:** ^1^School of Nursing, Faculty of Health, Dalhousie University, Halifax, NS, Canada.; ^2^Applied Research and Evaluation Consultant, Halifax, NS, Canada.; ^3^Faculty of Nursing, University of Montreal, Montreal, QC, Canada.; ^4^Montreal Heart Institute Research Center, Montreal, QC, Canada.; ^5^Faculty of Medicine and Health Sciences Research, University of Sherbrooke, Sherbrooke, QC, Canada.; ^6^Charles-Le Moyne – Saguenay–Lac-Saint-Jean Research Center on Health Innovations, Longueuil, QC, Canada.; ^7^Health Services Integration, Winnipeg Regional Health Authority, Winnipeg, MB, Canada.

**Keywords:** Health Services Research, Partnership Research, Co-production, Training, Integrated Knowledge Translation, Canada

## Abstract

Effectiveness in health services research requires development of specific knowledge and skills for working in partnership with health system decision-makers. In an initial effort to frame capacity-building activities for researchers, we designed a workshop on working collaboratively within the health system. The workshop, based on recent research exploring health system experience and perspectives on research collaborations, was trialed at the annual Canadian Health Services and Policy Research (CAHSPR) conference in May 2019. Participants reported positive evaluations of the workshop. However, further efforts should target health services researchers that may not be as motivated to develop skills in collaborative research. Additional attention to equipping researchers with the skills needed to work in partnerships is recommended, including approaches and materials that avoid oversimplification of complex challenges.

## Introduction

There are increasing expectations for engagement between researchers and knowledge users in the health system (eg, clinicians, decision-makers, policy-makers) in the prioritization, development, interpretation, and application of research evidence.^1,2^ Collaborative research approaches, such as co-production, co-design, engaged scholarship, and integrated knowledge translation^3^ aim to bridge the evidence to practice/policy gap by producing relevant research to address health system problems. Studies indicate several benefits to collaborative health services research, including improved quality of research,^4^ enhanced value for research among decision-makers,^5^ increased capacity among decision-makers for engaging in research,^5,6^ and more impactful and useful research findings.^4,7,8^

Collaborative research approaches blur the boundaries of research, policy, and practice domains to support researchers and health system decision-makers in working collaboratively to address complex healthcare problems.^9^ However, there are many challenges associated with building and maintaining effective collaborative partnerships. Research has shown that significant time is required to develop trusting, authentic relationships and there may be insufficient resources to support partnership development and maintenance.^10,11^ Other studies have found that researchers and health system decision-makers often have different values and priorities. This can lead to a mismatch of agendas and expectations, and may result in disagreement on the importance of the research findings and conflicting interpretations of what to do with the results.^4,5,10^ Consequently, there are often challenges with developing a study timeline that can meet the rigorous scientific process and respond to the fast-pace decision-making process in the health system.^4,5,10^

Our research team had previously examined the challenges associated with collaborative research from the perspective of health system decision-makers.^1,12^ While study participants echoed many challenges mentioned above, they also identified a lack of appropriate researcher preparation for engaging in collaborative health research. Effective health services research requires researchers to have specific knowledge and skills for working in partnership with health system decision-makers.^10,13^ However, most graduate training programs prepare students for traditional academic careers in health research.^14^ Thus, researchers often do not have the opportunity to learn how to build effective collaborative relationships with health system decision-makers.^10^ A recent review of websites of health research bodies and health system management also identified an absence of guidance on partnerships for health system leaders in working with research institutions, suggesting a need for initiatives to prepare trainees and researchers to engage in meaningful research-health system collaborations.^15^

As a first step in responding to this gap, we designed a workshop for Canadian health services researchers. We trialed the workshop at the annual Canadian Health Services and Policy Research (CAHSPR) conference held in Halifax, Nova Scotia, Canada, in May 2019. The purpose of this paper is to present a summary of the workshop activities and participant evaluation of its utility for health system researchers. Implications for future efforts to improve researcher preparation for working with the health system are also discussed.

## Methods

### Workshop Participants

CAHSPR is a national conference that attracts trainees, researchers, health system leaders, and patient partners from across Canada and globally. The 2019 conference theme was “When Research Meets Policy” and focused on how evidence shapes health policies and improves the lives of Canadians. All delegates of the CAHSPR conference were eligible to attend the pre-conference workshop and the registration cost was included in the conference fees.

### Intervention - Workshop Description

The workshop, titled “*Working Collaboratively within the Health System: Becoming an Effective Research Partner* ,” was based on our recent research^12,15^ and integrated the expertise of researchers working in embedded researcher roles. The workshop was sponsored by the CAHSPR Student Working Group and designed for current and recent graduate students and postdoctoral fellows in the field of health services and policy research. The workshop aimed to:

1. Summarize findings on barriers/challenges to research partnership from a health system perspective
2. Outline the research and interpersonal knowledge and skills that health system decision-makers are looking for in researchers
3. Provide practical guidance for establishing, managing, and supporting positive collaborations with health system decision-makers
4. Practice problem solving common partnership challenges.

The 4-hour workshop consisted of a series of interactive activities, including table discussions, PowerPoint presentations, case studies, polling activities using the Poll Everywhere^©^ platform,^16^ and large group discussions (Table 1). Designated table facilitators included seven Masters and PhD students from the CAHSPR Student Working Group. Responsibilities included reporting back a summary of the individual table discussions and recording notes which were shared with the workshop facilitators.

**Table 1:** Workshop Agenda

**Time**	**Agenda Item**	**Comments/Questions**
13:00	Introduction	Introduction activities 1. Table discussion: Why did you decide to sign up for this workshop? 2. Polling questions: Where do you live? What is your role?
13:25	**Activity 1:** What health system leaders are saying about research partnerships?	
13:25	(a) Presentation	This will be based on research findings and intended to provide an overview of current evidence of health system manager perspectives Focus will be on health systems/services research
13:50	(b) Group/table discussion Discussion questions: *1. What is your response to these findings based on your own experience (or expectations)?* *2. What skills and expertise do you feel is needed to engage with health system leaders in a research role? How well prepared do you feel?*	Objectives: To engage participants in reflecting on how findings relate to their personal experience To reflect on implications of findings for recommended preparation of health services researchers
14:15	(b) Synthesis/reflection	Facilitated discussion/brainstorming To include question and answer with presenters
	**Activity 2:** Case Studies for Establishing and managing effective partnerships	Objective: To apply general principles identified in introductory presentation to concrete situations and challenges
14:35	(b) Case study 1 Introduce case study (1) Explain Polling exercise (2) Respond to case study via poll (1)	
14:40	Large group reflection/discussion	Debrief on case study
15: 05	BREAK	Break will occur at approximately 3:00 p.m. at a natural breaking point
15:20	Case studies 2 and 3	Divide the case studies between the groups
	Table discussion	Discussion to focus on response to case study questions
15:35	Large group debrief	
15: 55	Case study 4 Similar format to case study 1	
16:05	Large group reflection/discussion	
16: 20	Case study 5 Similar format to case study 1	
16: 25	Large group reflection/discussion	
16:40	**Activity 3:**Next Steps and Evaluation	
	(a) Large group brainstorming activity	Question for brainstorm: 1. What changes would you suggest to your academic preparation for working with the health system? 2. Based on our conversation today, what practical guidance/resources would be helpful to support academics in their partnerships with the health system
	(b) Wrap Up and Evaluation	Both written evaluation and closing polling slide(s) for closing comments (these will be introduced at the beginning of the workshop)

#### Activity 1: Research Findings Presentation

To set the stage, we presented a 25-minute PowerPoint summary of the findings from our national study,^12^ with specific emphasis on findings of relevance to health services researchers. Following the presentation, participants discussed their response to the research findings at their respective tables, and provided a summary to the larger group. Objectives of this activity were to reflect on: (*i*) The relation of findings to their experience or expectations; and (*ii*) The implications of findings for the preparation of health services researchers.

#### Activity 2: Case Studies

A main component of the workshop was a series of 5 case studies (Supplementary file 1). The overarching objective of the case studies was to apply general problem-solving skills to concrete situations and challenges. The CAHSPR Student Working Group contributed to identification of potential case study topics and the workshop facilitators built the case study scenarios (Table 2) based on a mix of actual events from the facilitators’ experiences and research findings. All case scenarios were masked for the purpose of the workshop.

**Table 2:** Case Study Scenarios and Key Learning Points

**Case Study Title**	**Format**	**Key Learning Points**
1. Getting established within a health system organization	Online polling with multiple choice	• Need for researcher humility • Work demands within the current health system context
2. Arranging student placements	Table and large group discussion	• Need for greater academic responsiveness to the health system context
3. Building a collaborative team	Table and large group discussion	• Differing priorities Importance of preventing misunderstandings rather than troubleshooting • Need to ensure health system contributions are recognized and compensated
4. Managing a problem within an agreed-upon collaboration	Online polling with multiple choice	• Importance of researcher humility • Authentic collaboration • Importance of determining who needs to be involved in the partnership activity
5. Rigor vs. relevance	Online polling with multiple choice	• Challenges of balancing desire for a rigorous study design with health system needs and resources • Differences in system-driven versus researcher-driven research • Need to ensure that methods reflect the question of concern to the organization

#### Activity 3: Next Steps and Evaluation

The final activity included a brief outline of suggested practical guidance for researchers and a large group brainstorming activity on next steps. We asked participants to suggest needed changes to their academic preparation for working with health system decision-makers and to identify practical guidance/resources that may be helpful. To evaluate the workshop, we asked participants to provide a one-word descriptor of the workshop via the Poll Everywhere^©^ software and complete an anonymous evaluation form to provide additional insights on the workshop.

### Data Collection

At the beginning of the workshop, participants were informed of the objective of the workshop and that we would be evaluating its usefulness for future planning. Table facilitators recorded summaries of small group discussions and shared their notes with the workshop facilitators. During the large group discussions, the workshop facilitators recorded the discussion on a flip-chart. For the polling activities, all participant responses were collected via the Poll Everywhere^©^platform. We collected evaluation feedback with the Poll Everywhere^©^ platform and an anonymous 9-item hard-copy evaluation form. As this was a workshop evaluation activity, ethics review was not sought and only general, aggregated summaries of discussions are reported.

### Data Analysis

Table facilitator notes and flip-chart notes were collated into a single document. Activity 1 discussion was summarized narratively. A conventional content analysis approach was used to analyze the case study descriptions (Activity 2). One facilitator (CEC) reviewed the document and categorized similar statements into overarching key themes of discussion: these themes were reviewed by SB. Polling findings were analyzed descriptively and integrated with the discussion themes. Workshop evaluation data (Activity 3) were analyzed with frequency counts and the open-ended questions were summarized narratively. At this point, a draft report was circulated to all table facilitators for review, discussion and input to ensure that findings accurately reflected experience of discussions at all tables.

## Results

### Participant Characteristics

**Figure 1:**
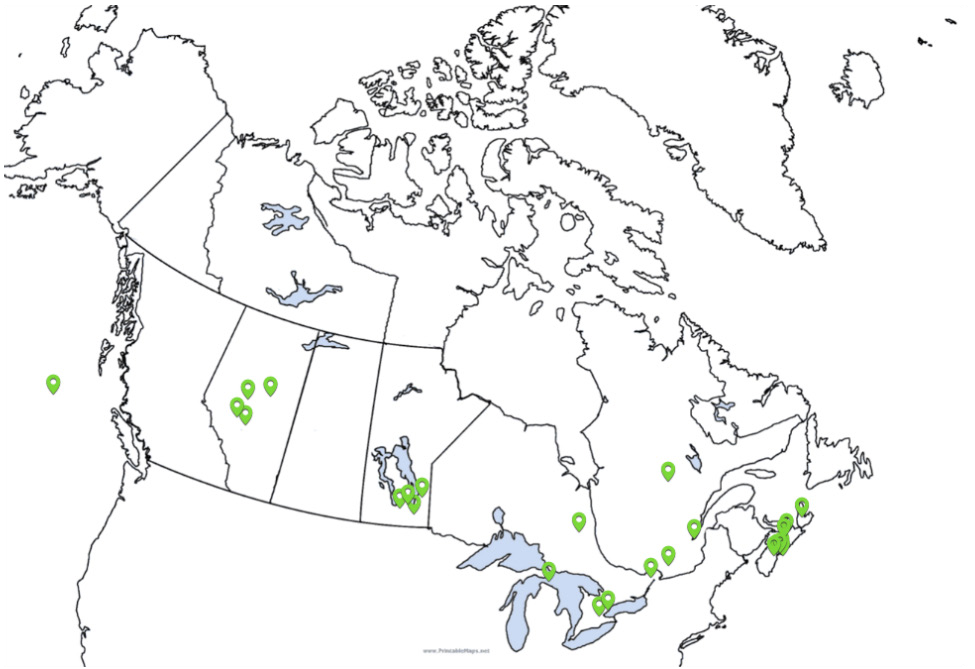
Geographic Distribution of Workshop Participants.

Thirty-five participants attended the workshop (including 7 table facilitators). Of those participants who completed the polling questions (n = 24), the majority were trainees in health services research, including Masters students (n = 8), PhD students (n = 3), and postdoctoral fellows (n = 3). Eight participants were researchers in an embedded position in the health system (n = 5) or in a university (n = 3). One knowledge user and one patient advisor also attended. Participants came from five provinces across Canada (ie, Alberta, Manitoba, Ontario, Quebec, and Nova Scotia), and there was one international participant (Figure 1).

### Activity 1: Research Findings Presentation

#### Motivations for Attending

Several participants described past challenges when partnering with the health system as a reason for attending. These challenges included experiencing competing priorities and agendas, lack of time, and a continuous need for negotiation. In addition, many attended the workshop to learn more about the “mechanics” of health system partnerships, including how to engage and collaborate with different stakeholders, manage ongoing collaborations, and partner more effectively with stakeholders with different agendas. Participants hoped to gain “tips and tricks” for conducting collaborative research and solving potential research-health system tensions. Finally, participants stated that they were motivated to attend due to their interest in knowledge translation and answering research questions important to the health system.

#### Participant Response to Research Findings Presentation

Participants stated that the results from the national study resonated with their experiences. Several tables discussed the disconnect between researchers and health system leaders as the two groups work with different “currencies” (eg, publications, quality indicators), as well as competing demands, timeframes, and expectations. Participants reiterated the importance of being clear about whether the research is driven by system or researcher interest. They emphasized the need for time to understand multiple perspectives and develop authentic partnerships, and the need for clarity and transparency when articulating expectations for collaborating on a common goal. Participants proposed several skills they felt were needed to engage with health system leaders in a research role, including strong communication and interpersonal skills; readiness and willingness to engage in different types of partnerships; generalist expertise in research methodologies; flexibility; and reliability.

#### Activity 2 – Case Studies: Key Discussion Themes

#### Health System Complexity

A common thread discussed throughout the case studies was the complexity of the health system and work demands within the current health system context. Participants recognized that time is needed to establish relationships and understand organizational culture; however, organization staff may feel overwhelmed with time pressures and competing priorities. Facilitators stressed the importance of ensuring an understanding of both issue and context before making suggestions; they also reflected on potential frustrations among health system decision-makers about lack of researcher awareness of the context.

#### Authentic Partnerships

Participants recognized that true partnership does not refer to superficial consensus; it should focus on engagement and respect diverse expertise and perspectives. For example, when designing a research study, it is important to work with health system decision-makers to clarify what outcomes are most important to the group and how they plan to use the findings. Further, participants noted that not all research must be “partnership” research. Facilitators stressed that in some cases, it may only be that the researcher is interested in site access. They noted that health system decision-makers may be supportive of student learning and research in general and will likely try to accommodate requests if appropriate. By being honest and transparent, researchers can aim to align their requests with the workload of health system decision-makers. However, participants stressed that it can be challenging to manage the tension between valuing honesty and transparency, and working with organizational stakeholders who wish to amend research projects to address local needs.

While discussing case study 4, facilitators reflected on experiences of inauthentic collaboration (eg, scheduling meetings around availability of the researcher without considering the competing time demands of the health system decision-makers; failing to involve the key organizational personnel who had in-depth understanding of the data). As a result, the appropriate health system decision-makers may not have the opportunity to participate in discussion or provide needed guidance. Facilitators reiterated the need for key organizational members to be engaged initially and throughout the process in order to understand and benefit from internal expertise. It was noted that while sometimes findings may not be what was hoped, this was an issue that should be addressed through adequate project planning. Participants discussed the importance of keeping stakeholders informed of emerging study results throughout the project. Misunderstandings, in addition to potentially increasing project costs, can create stress and distrust, with long-term impacts on partnerships.

#### Challenges With Collaborations

Several of the case studies highlighted challenges with establishing and sustaining collaborations. In case study 2, participants outlined potential reasons for difficulties in arranging student placements, including unclear expectations, lack of resources (time, personnel, space, financial), previous negative experiences, privacy and confidentiality concerns, and a lack of understanding of the value of the research collaboration. Facilitators reinforced participants’ observations that short-term placements can be particularly problematic as it often takes several months of orientation before a student is ready and able to work unsupervised. Summer placements may pose additional issues due to staff vacation time. Health system decision-maker workload may prevent provision of needed supervision, and there may be challenges with identifying an appropriate project for a student to work on within a specific timeframe. In case study 3, participants identified additional potential reasons for difficulties with collaborations: inefficient and ineffective meetings; lack of incentives for staff participation; failure to involve appropriate partners; and ineffective email practice. Facilitators also discussed the impact of failure to clearly negotiate roles, responsibilities, work plans, backup plans, and deadlines before the project started.

#### Potential Solutions and Strategies

Each case study led to a discussion on potential strategies to facilitate successful health system-researcher collaborations. Participants identified the need to address expectations before the project begins, which may include developing contracts or joint documents that outline clear roles and expectations. Facilitators commented on the importance of being open to changing expectations of how the consultation or partnership will occur, as the situation may be continually evolving. Further, participants suggested restructuring the meeting approach (eg, establish small working groups, develop clear meeting agendas, identify and prioritize participation of key partners, synthesize information to critical key messages) and consider communication and planning options other than meetings. Participants also identified structural changes that may improve collaboration, including funding and support for organizational involvement, project management resources, involving the organization from the outset, identifying internal champions, and ensuring key players are included early. Universities could provide more support to researchers and help establish clear communication channels; research funders should consider the time and resource demands placed on healthcare staff and develop strategies to compensate their contribution; and health system organizations could consider “relationship broker roles” to facilitate project development while supporting researchers’ learning about organizational culture and structure.

### Activity 3: Next Steps and Evaluation

**Figure 2:**
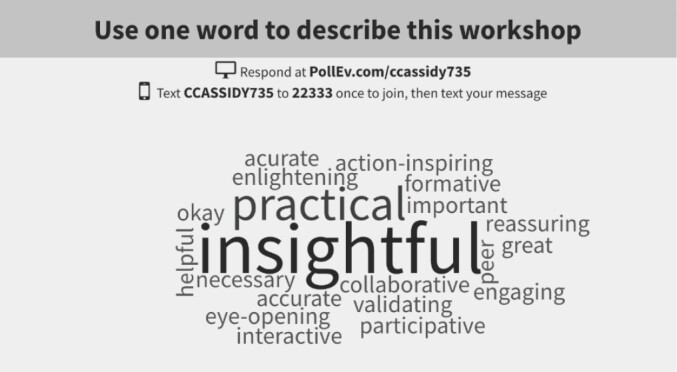
One-Word Evaluation of the Workshop.

Participants identified the need to incorporate more leadership development and change management during their academic training. They suggested internships that focus specifically on “how to” do collaborative research. They also questioned the balance between researcher- and organizational-driven collaborative research. Participants generally felt that collaborative research is dominated by a traditional research paradigm with its design, goals, processes, and outcomes. There seems to be interest in investigating the characteristics of collaborative research led by organizational stakeholders, and what that would imply in terms of skills needed among researchers. Moving forward, participants suggested the need for funding resources for evaluation or system-oriented work within the health system, and opportunities for health system decision-makers and research trainees to build collaborative research projects together. Figure 2 illustrates the feedback provided with the one-word evaluation activity, with larger text indicating a greater frequency that the word was submitted. Twenty-three participants completed the workshop evaluation form. The majority of participants (95%) felt the workshop met their expectations quite well (n = 12) or to a great extent (n = 10), identifying case studies as the most interesting part of the session.

## Discussion

This workshop was designed as an initial step to better prepare researchers to work effectively in research partnerships with the health system. Formal evaluation activities, combined with analysis of participant response to presentations and case studies, assessed the extent to which the workshop activities resonated with participants’ experience, and may be helpful for larger educational initiatives. In this section, we reflect on what was learned from the workshop and implications for further action.

### Participant Experience With Collaborative Health Research

Participants were clearly motivated to learn more about collaborative research. Many brought practical experience, and overall their comments were thoughtful and insightful. Participant feedback confirmed that the key issues addressed in the case studies were relevant, and reinforced research findings of gaps in preparation for collaborative work. Differences in the program of study (eg, Masters, PhD, postdoctorate) did not seem to be as important as experience with the health system and commitment to partnership approaches.

The case studies were not difficult for these participants; in fact, several were looking for more depth. It may be that attendees self-selected based on an interest and awareness of the need for more skill development. If so, appropriateness of the resources and approach for general use would need to be tested. Even more importantly, if it is indeed the case that attendees were already among the most aware and committed to the issue (suggested by participants comments that they were looking to have their experiences validated), this highlights the limitations of relying on optional workshops to develop key partnership skills and insights. We were left questioning whether we were “preaching to the converted;” Perhaps researchers who would most benefit from such interventions are those who may not recognize the need for partnership skills.

### Collaborative Health Research Training

Moving forward, it would be useful to consider how best to target health services researchers who are not currently engaged in collaborative health research. Collaborative training initiatives have recently been developed to address some of the gaps in collaborative research skills. For example, the Canadian Institutes of Health Research (CIHR) Health System Impact Fellowship was designed to modernize doctoral and postdoctoral training with specific enriched core competencies that target collaborative research skills.^17^ Similarly, in the United States, Academy Health developed the Delivery System Science Fellowship to provide experiential learning and professional development opportunities for postdoctoral trainees.^18^ While such programs have the potential to enhance trainees’ collaboration skills, it is important not to rely solely on optional training opportunities, and to ensure that rigorous evaluation enables assessment of the extent to which both health system personnel and trainees find these placements useful. If we are to achieve the many benefits of research collaboration, learning how to be an effective research partner cannot be treated simply as an optional “add on” only to be pursued by researchers and trainees with an interest in the topic. We argue that all knowledge and skills, including these core professional skills, should an integral, mandatory component of the core academic curriculum.

### Recommendations for Building Collaborative Research Capacity

While we have experience with only one workshop conducted with a motivated group of workshop participants, there are some additional suggestions we propose for similar initiatives. A few participants felt that the researcher perspective was missing from this conversation. We intentionally focused on health system perspectives as, to date, most research in the area has focused on researcher perspectives.^8,10^ In development of a comprehensive ‘curricula,’ more extensive coverage of the similarities and differences of various perspectives (including that of researchers employed by and within the health system) would be useful. Further, curriculum development initiatives could build on existing resources, such as the Researcher Development Framework,^19^ which has a specific domain focused on working with others to engage, influence, and impact, but lacks specificity to health system research partnerships.

Further, many participants were looking for concrete “take aways” (tangible strategies, “dos” and “don’ts,” information sheets, etc). While we support the need to develop more resources, we would also caution about oversimplifying a complex undertaking. Collaborative research is primarily relational work focused on understanding a different worldview and working together to solve a problem. Due to its complexity, there may be risks in attempting to distill guidance into simple resources such a “how-to” checklist. In addition, we would propose that distribution of overly simple resources may suggest that collaborative research partnerships may not require significant time, effort, and self-reflection.

## Conclusion

This paper describes findings from a workshop focused on enhancing researcher knowledge and skills for working in partnership with health system decision-makers. Our discussion contributes to the growing literature on the importance of collaborative health research and confirms the need for tailored capacity-building initiatives. While participants reported positive evaluations of the workshop and confirmed the importance of this topic, further efforts should target health services researchers that may not be as motivated to develop specific skills in collaborative research. Additional attention to equipping researchers with the skills required to work in partnerships is needed, including approaches and materials that avoid oversimplification of complex challenges. These efforts will assist in building capacity amongst the research community for establishing and maintaining effective health system-research partnerships.

## Acknowledgements

We wish to acknowledge the support of the CAHSPR Student Working Group in the development and facilitation of this workshop. This workshop was supported by the CIHR-funded Foundation Grant (FDN #142337) “*Moving Knowledge into Action for more effective practice, programs and policy: A research program focusing on integrated knowledge.* ”

## Ethical issues

Not applicable.

## Competing interests

Authors declare that they have no competing interests.

## Authors’ contributions

CEC, SB, and IB conceptualized the manuscript. CEC and SB prepared the initial manuscript draft. SB, GF, ÉCB, and IB provided critical revision of the manuscript. All authors read and approved the final manuscript.

## Authors’ affiliations

^1^School of Nursing, Faculty of Health, Dalhousie University, Halifax, NS, Canada. ^2^Applied Research and Evaluation Consultant, Halifax, NS, Canada. ^3^Faculty of Nursing, University of Montreal, Montreal, QC, Canada. ^4^Montreal Heart Institute Research Center, Montreal, QC, Canada. ^5^Faculty of Medicine and Health Sciences Research, University of Sherbrooke, Sherbrooke, QC, Canada. ^6^Charles-Le Moyne – Saguenay–Lac-Saint-Jean Research Center on Health Innovations, Longueuil, QC, Canada. ^7^Health Services Integration, Winnipeg Regional Health Authority, Winnipeg, MB, Canada.

## Supplementary files

Supplementary file 1 contains 5 case studies.
