# Supplementary material for: How to Work Collaboratively Within the Health System: Workshop Summary and Facilitator Reflection
Source: Int J Health Policy Manag. 2019 Dec 8;9(6):233–9. doi: 10.15171/ijhpm.2019.131 (PMC7382904; doi:10.15171/ijhpm.2019.131)
Supplement: Supplementary file 1 — contains 5 case studies. [file ijhpm-9-233-s001.pdf]

Supplementary file 1

Case Studies (For Participant Distribution)

Case Study 1: Getting Established Within an Organization

You have just been invited to a health region meeting focused on chronic disease management (CDM), which has been identified as a major organizational priority. This is your first opportunity to meet with many of those you hope to work with as a researcher and you are hoping this meeting will provide the first step in you forming research partnerships. The discussion is wide ranging, and does not seem to reflect the latest research in this field. As CDM an area that you are knowledgeable about, you are confident you can make a contribution.

What would be the best action for you to take in order to demonstrate your value?

Case Study 2: Arranging Placements

Your university department is participating in 4 month summer placement program for research trainees. It feels that offering Masters and PhD students with specialization in health services research to health organizations at the small price of a summer stipend will not only be of great benefit to students but also enable the health system to accomplish tasks it would not otherwise be able to take on. The major health provider in your area has, however, firmly declined the offer of student internships.

a) What might some of the reasons for this be?

b) What strategies would you propose to encourage greater health system participation?

Case Study 3: Building a Collaborative Team

You have been successful in receiving support from a local health organization to conduct a study on their site and using their data. There is very positive support for the research as it addresses a question of concern to several program areas. You are now at the stage of refining the research question(s), and designing the research project. You recognize that you need to have the active involvement of key managers and staff, as the research will require access to confidential data, and

is integrated into service delivery activities. As a result, you have worked with supporters within the organization to determine who needs to be involved, and they have responded positively both to the research proposal and to the invitation to be part of the research team. However, you are finding that it is very difficult to get good attendance at meetings; often there are last minute cancellations; and - even when there is a time-sensitive issue that needs quick action - it is difficult to get a response. As a result your project is running behind and you are concerned that you may miss several important 'windows' for action.

What might explain the difficulties you are experiencing?

How might you best respond to these difficulties?

What other changes (in planning, in funding, in structures) may have helped avoid these challenges?

Case Study 4: Managing a Problem Within an Agreed-Upon Collaboration

A young researcher in your department has been placed as the lead on a patient safety study with the health region. The department and the region have had several successful collaborations covering many years. There is a lot of regional support for the research question, and the research team has been given access to internal data. The agreement between the department and the region is that a confidential draft report must be circulated to senior management before the before publicly releasing the report. An advisory body of key clinical and senior management staff has been established, and meets 3 times over the year to complete the report.

When the draft report is released internally, there is a great deal of distress and many criticisms about the methodology. You learn from one colleague that a meeting of clinicians was held that resulted in table pounding and insults, and from another that the researcher is feeling so stressed that he is taking stress leave.

How might you interpret this sequence of events? What action would you recommend?

Case Study 5: Rigour Vs. Relevance

As part of your postdoctoral training, you are embedded in your province's Department of Health. You are excited to apply your advanced expertise in research methodologies to address complex issues in the real world. During your first few months, you work closely with your decision-maker partners to identify a research question that meets both the needs of the organization and your learning needs as a postdoctoral fellow. The question you all agree on is to determine the impact of a healthy lunch program in all elementary schools across the province. You believe a cluster randomized controlled trial design would be the best methodology to answer this research question; however, your partners are not in favour of a trial design because of the time and resources needed.

What would be the most effective action that would maintain rigour while conducting research useful to departmental staff?
